# Association of norepinephrine transporter methylation with in vivo NET expression and hyperactivity–impulsivity symptoms in ADHD measured with PET

**DOI:** 10.1038/s41380-019-0461-x

**Published:** 2019-08-05

**Authors:** H. L. Sigurdardottir, G. S. Kranz, C. Rami-Mark, G. M. James, T. Vanicek, G. Gryglewski, N. Berroterán-Infante, A. Kautzky, M. Hienert, T. Traub-Weidinger, M. Mitterhauser, W. Wadsak, A. M. Hartmann, M. Hacker, D. Rujescu, S. Kasper, R. Lanzenberger

**Affiliations:** 1 Department of Psychiatry and Psychotherapy, Medical University of Vienna, Vienna, Austria; 2 Department of Rehabilitation Sciences, The Hong Kong Polytechnic University, Hung Hom, Hong Kong, China; 3 Department of Biomedical Imaging and Image-guided Therapy, Division of Nuclear Medicine, Medical University of Vienna, Vienna, Austria; 4 Ludwig Boltzmann Institute Applied Diagnostics, Vienna, Austria; 5 Center for Biomarker Research in Medicine CBmed, Graz, Austria; 6 Department of Psychiatry, University of Halle, Halle, Germany

**Keywords:** Genetics, ADHD, Neuroscience

## Abstract

Attention deficit hyperactivity disorder (ADHD) is a common neurodevelopmental disorder with a robust genetic influence. The norepinephrine transporter (NET) is of particular interest as it is one of the main targets in treatment of the disorder. As ADHD is a complex and polygenetic condition, the possible regulation by epigenetic processes has received increased attention. We sought to determine possible differences in NET promoter DNA methylation between patients with ADHD and healthy controls. DNA methylation levels in the promoter region of the NET were determined in 23 adult patients with ADHD and 23 healthy controls. A subgroup of 18 patients with ADHD and 18 healthy controls underwent positron emission tomography (PET) with the radioligand *(S,S)-*[^18^F]FMeNER-D_2_ to quantify the NET in several brain areas in vivo. Analyses revealed significant differences in NET methylation levels at several cytosine–phosphate–guanine (CpG) sites between groups. A defined segment of the NET promoter (“region 1”) was hypermethylated in patients in comparison with controls. In ADHD patients, a negative correlation between methylation of a CpG site in this region and NET distribution in the thalamus, locus coeruleus, and the raphe nuclei was detected. Furthermore, methylation of several sites in region 1 was negatively associated with the severity of hyperactivity–impulsivity symptoms. Our results point to an epigenetic dysregulation in ADHD, possibly due to a compensatory mechanisms or additional factors involved in transcriptional processing.

## Introduction

Presented by symptoms of hyperactivity, inattention, and impulsivity, attention deficit hyperactivity disorder is (ADHD) one of the most frequent neurodevelopmental disorders in children that persists into adulthood in ∼30% of the cases. While the exact underlying neurobiology of ADHD remains elusive, there is a general consensus that genetics contribute significantly to the etiology of the disorder, with an estimated heritability factor of 0.77 [1]. With many genes being investigated and only a few risk genes having been identified, the complex mechanism of the disorder is elucidated and suggests a role for gene–environment interactions [2].

Among the several genes investigated in ADHD the SLC6A2 gene which encodes for the norepinephrine transporter (NET) is included. It regulates norepinephrine homeostasis and is responsible for the reuptake of norepinephrine (and dopamine in prefrontal regions) into the presynaptic neuron [3]. It is implicated in ADHD as common medication such as methylphenidate (MPH) target the dopamine and norepinephrine transporters [4]. Several single nucleotide polymorphisms (SNPs) within the NET gene have been investigated in ADHD and some have been associated with the disorder and related behavioural phenotypes [5–7]. We previously did not detect differences in the NET binding potential between patients and controls [8], while on the other hand we observed genotypic differences in the NET binding potential in the thalamus and cerebellum between adults with ADHD compared with healthy controls (HC). Furthermore, we detected an association between hyperactivity–impulsivity symptom scores and cerebellar NET binding that was genotype dependent in adult ADHD patients [9]. Therefore, epigenetic mechanisms are potentially involved in the pathophysiology of ADHD.

In recent years, the importance of DNA methylation has received increased attention as a possible modulator in psychiatric disorders in addition to the influence of genetic polymorphisms. DNA methylation is an epigenetic mechanism in which a methyl group is added to cytosine in cytosine–phosphate–guanine sites (CpG). This process can directly affect the activity and function of a gene without altering the DNA sequence: the methylation of these sites interferes with the binding of transcription factors and on the other hand of methyl-binding proteins can repress gene expression [2, 10].

A few investigations have examined the role of DNA methylation in ADHD. The study by van Mil et al. detected lower DNA methylation profiles of several genes at birth to be correlated with ADHD symptoms at the age of 6 years [11]. Another study investigated DNA methylation levels of dopamine transporter (SLC6A3) with methylphenidate response in children with ADHD. They report a negative correlation between methylation levels and response to treatment, namely oppositional and hyperactive-impulsive symptoms, indicating that lower levels of methylation were associated with greater symptom improvement [12]. We know of only one study that investigated the SLC6A2 methylation in ADHD. In that study, the authors examined an abundance of genes, including the SLC6A2 in boys with ADHD and found that SLC6A2 methylation was associated with Cue-P3 task which is related to the posterior attention network [13].

Given our previous findings on the genetic influence on NET availability and behavioral symptoms, we sought to extend and complement our previous investigation in order to gain more insight by establishing whether any potential influence or interaction of epigenetic factors is present. With that we sought to assess if interaction of polymorphisms and DNA methylation potentially affect behavior and brain function. Our first aim was to test whether there is a difference in DNA methylation levels of CpG sites in the NET promoter between patients with ADHD and HC. Secondly, effects of candidate SNPs on the DNA methylation levels were explored. Thirdly, we assessed any potential associations between behavioural symptoms and NET methylation levels. Finally, we tested whether observed differences in methylation profiles translate to differential expression levels of the NET measured by PET.

## Methods

In total 23 adult ADHD patients (age ± SD: 32.2 ± 10.9, 16 males) and 23 HC (age ± SD: 30.9 ± 10.6, 16 males) of which data have been published previously participated in the study [8, 14]. Subgroup analysis for association with NET binding potential (BP_ND_) and for testing of potential influence of SNPs included 18 adult patients with ADHD (age ± SD: 30.3 ± 10.5, 11 males) and 18 HC (age ± SD: 29.9 ± 10.5, 11 males). Subjects were recruited through the ADHD outpatient clinic at the Department of Psychiatry and Psychotherapy, Medical University of Vienna and via advertisement as previously published [8, 14]. Patients had been free from any psychopharmacological treatment at least 6 months prior to study inclusion.

Subjects underwent physical examinations and were tested for current substance use with a urine test. Inclusion criteria demanded for patients to have history of symptoms in childhood and a current diagnosis of ADHD. Subjects were interviewed using the Conners‘ Adult ADHD Diagnostic Interview for DSM-IV (CAADID, Conners, 1999), Conners‘ Adult ADHD Rating Scale Investigater-Screen Version (CAARS-Inv:SV), Conners‘ Adult ADHD Rating Scale: Observer-Screen Version (CAARS-O:SV), and the Conners‘ Adult ADHD Rating Scale: The Self-report Screening Version (CAARS-S:SV). Subjects were excluded if they had any comorbid DSM-IV Axis I and II disorder as determined by the Structural Clinical Interview for DSM-IV. Written consent was aquired from all participants and they were financially reimbursed for their participation. The Ethics Committee of the Medical University of Vienna approved this study.

### Selection of single nucleotide polymorphisms and genotyping

Four SNPs were included based on our previous publication: rs28386840, rs2242446, rs40615, and rs15334 [9].

Procedures were performed as previously described [9]. In short, 9 ml of blood from each subject was collected in EDTA blood tubes. Isolation of DNA was done using the QiaAmp DNA blood maxi kit (Qiagen, Hilden, Germany). Genotyping was performed using the iPLEX assay on the MassARRAY MALDI-TOF mass spectrometer. Allele specific extension products were selected and genotypes assigned by Typer 3.4 Software (Sequenom, San Diego, CA, USA). Quality criteria (of individual call rate >80%, SNP call rate >99%, and identity of genotyped CEU trios (Coriell Institute for Medical research, Camden, NJ) with HapMap database >99%) were applied and met.

### Bisulfite sequencing and definition of methylation regions

The location of the selected CpG islands is as follows: chr16: 55655473–55656461 (see Supplemental Fig. 1). Location and definition of promoter regions was based on previous publications demonstrating these regions to be transcriptionally important [15–17]. In order to assess the methylation levels at individual CpG sites, the following three regions were bisulfite sequenced and are comprised of the following CpG sites: CpG1, CpG2.3, CpG4, CpG5, CpG6, CpG7.8, and CpG11.12 (region 1), CpG5.6.7, CpG8.9.10, CpG11.12, CpG25, CpG26.27, and CpG54.55 (region 2), and lastly, CpG1.2, CpG2.3, CpG7, CpG8.9, CpG10, CpG11, and CpG12 (region 3) [16, 17].

Detailed protocol of the DNA methylation design and profiling using EpiTYPER is described by Suchiman et al. [18]. In short, around 100 ng of genomic DNA was converted into bisulfite using EZ-96 DNA methylation kit, Shallow-Well Format (ZYMO Research). This was followed by PCR amplification. The primers used for PCR amplification of regions are listed in Supplemental Table 1. Step down PCR reaction using 20 ng of bisulfite converted DNA was performed as by protocol starting with 15 min at 95 °C, followed by 4 cycles at 20 s at 95 °C, 30 s at 65 °C, and 1 min at 72 °C, thereafter 4 cycles: 95 °C for 20 s, 58 °C for 30 s, and 70 °C for 1 min. Last, 38 cycles as described: 20 s at 95 °C, 30 s at (AT)°C for 3 min, and at 72° for 1 min. The final extension was carried out at 72 °C for 3 min and cooled down to 4 °C. To check for the generation of PCR products, selected samples were run on a 1.5% Agarose gel. Following dephosphorylation of unincorporated dNTPs, PCR products were cleaved into smaller fragments using the MassCleave reaction at 37 °C for 3 h (Sequenom). After removal of excess ions, 15–20 nl of each sample were spotted onto a SpectroCHIP II-G384 and analyzed with the Epityper 1.2 (Agena Bioscience).

Quality control included the following criteria: sample call rate >50%, CpG call rate >85%, and duplicate values with stdev <0.1). In order to confirm and increase our call rates, a new measurement was done using the same method. With that we were able to confirm our previous results as well as increase our call rates with the addition of 13 CpG sites. All sites not fulfilling the criteria were excluded from further analysis.

The resulting data from the mass spectrometer was preprocessed using the EpiTYPER Analyser. A period between number annotated at the same CpG illustrates that the sites occur within the same fragment. Average methylation was calculated for each of the three regions investigated.

### Positron emission tomography (PET)

Subjects underwent PET (General Electric Medial Systems, Milwaukee, WI, USA) scans at the Department of Biomedical and Image-guided Therapy, Division of Nuclear Medicine at the Medical University of Vienna applying the tracer *(S,S)-*[^18^F]FMeNER-D_2_ [19]. Detailed information regarding the scans have been described previously [8]. A retractable ^68^Ge rod source for tissue attenuation correction was performed prior to the dynamic emission scan, during a 5-min transmission scan and acquired in 3D mode. The acquisition of data started at 120 min after a bolus i.v. injection of 4.7 MBq/kg body weight (ADHD patients: 393 ± 95 MBq, HC: 384 ± 61 MBq; *p* > 0.05, *t*-test) of *(S,S)*-[^18^F]FMeNER-D_2_. The mean value of the specific radioactivity of *(S,S)*-[^18^F]FMeNER-D_2_ was 537 ± 383 GBq/μmol (ADHD patients) and 473 ± 218 GBq/μmol (HC), (*p* > 0.05, *t*-test). Series of six consecutive time frames each lasting 10 min in an interval of 120–180 min after tracer bolus application was performed to measure radioactivity in the brain. The collected data was reorganized in volumes consisting of 35 transaxial sections (128 × 128 matrix) using an iterative filtered back projection algorithm (FORE-ITER) with a spatial resolution of 4.36 mm full width at half-maximum 1 cm next to the center of the field of view. Magnetic resonance (MR) images from subjects taken on a 3 Tesla Philips scanner (Achieva) using a 3D T1 FFE weighted sequence, yielding 0.88 mm slice thickness and in plane resolution of 0.8 × 0.8 mm were used for coregistration [8].

### Data preprocessing and quantification of norepinephrine transporter

Information on data preprocessing and the quantification of the NET is described in detail elsewhere [8]. In short, individual time frames of the dynamic PET scan were readjusted to the mean of frames with no head motion, determined by visual inspection. The readjusted images were then coregistered to each subjects MRI scan using a mutual information algorithm in SPM8 (Wellcome Trust Centre for Neuroimaging, London, UK: http://www.fil.ion.ucl.ac.uk/spm/). The caudate is considered devoid of NET [20] and was therefore used as the reference region for the parametric images of NET BP_ND_. The caudate was manually delineated on individual MRIs using PMOD image analysis software, version 3.1 (PMOD Technologies Ltd, Zurich, Switzerland, www.pmod.com). NET quantification was calculated according to Arakawa et al. [21]. BP_ND_ was calculated as the ratio between the area under the time-activity curve of the target region and the area under the time-activity curve for the reference region minus 1. An integration interval of 120–180 min was applied. The caudate was manually delineated on individual MRIs using PMOD image analysis software, version 3.1 (PMOD Technologies Ltd, Zurich, Switzerland, www.pmod.com). The developed transformation matrices were applied to the coregistered parametric images and then warped into MNI standard space.

### Regions of interest (ROIs)

The selection of brain ROIs was based on regions containing high expression of the NET [20, 22] as well as target regions in behavioral control [23]. Those regions included the thalamus, locus coeruleus, putamen, cerebellum, and the raphe nuclei. The ROI NET BP_ND_ was extracted from the Hammer Maximum Probability Atlas (Hammers, et al. 2003) and through manual delineation on the MNI T1 single-participant brain. The *(S,S)-*[^18^F]FMeNER-D_2_ radioligand introduces a potential bone spill over and hence cortical regions were excluded from the analysis. So far, the cause of the observed spillover remains unresolved. In in vitro experiments, defluorination and subsequent binding to bone could not be confirmed. Possibly, some other metabolic degradation route is responsible for this phenomenon [24].

### Statistical analysis

Descriptive parameters were computed and NET methylation levels were assessed for normality using the Shapiro–Wilk test. In case of deviation from normality, Mann–Whitney was computed to test for differences between study groups.

Effects of group (ADHD patients vs HC) on methylation levels were tested using linear mixed model using the average mean of methylation levels from each region, as well as individual CpG sites methylation values as the dependent variables. Potential confounding factors, such as previous medication status, age, and sex were accounted for and excluded if rendered insignificant. The model tested for main effects and any possible interactions between group and CpG sites on methylation. If rendered significant, post hoc analysis included Mann–Whitney tests and *t*-test in case of normality. Effects of SNPs (homozygous major vs minor allele) and group (ADHD vs HC) on binding potential and behaviour (see Supplement Page 2, Supplemental Table 2, Supplemental Figs. 2, 3), as well as methylation levels were also tested for using genotype (major vs minor allele), group (ADHD vs HC) as fixed factors and binding potential and methylation levels as the dependent variables.

Potential association of NET BP_ND_ and methylation levels were examined using a linear mixed model with a stepwise backward elimination procedure. The association of behavioral scales and methylation levels were investigated using Pearson correlation in patients only. Further regression analysis tested the combined effects of genotypes and methylation levels on binding potential and behavioral scales.

SPSS version 22.0 (IBM Corp. Released 2013. IBM SPSS Statistics for Windows, Armonk, NY: IBM Corp) was used for the analyses. The significance level was set at *p* < 0.05 and corrected for multiple comparisons using the Benjamini–Hochberg correction [25].

## Results

### Demographic characteristics

Demographic information of subjects is provided in Table 1. Demographics for the subgroup analysis is provided in Table 2. No difference of either age or sex was detected between groups.

**Table 1:** Demographics of total sample

	Patients with ADHD (*n* = 23)	Healthy controls (*n* = 23)
Age	30.9 ± 10.6	32.2 ± 10.9
Sex (male/female)	16/7	16/7
Conners’ Adult ADHD Rating Scale—Total score	35.91 ± 7.76*	0.84 ± 1.49*
Conners’ Adult ADHD Rating Scale—Hyperactive/impulsive	18.64 ± 5.08*	0.40 ± 0.83*
Conners’ Adult ADHD Rating Scale—Inattention	18.14 ± 4.53*	0.44 ± 0.84*

An asterisk indicates the statistically significant regions

**Table 2:** Demographics of subgroup analysis sample (PET sample)

	Patients with ADHD (*n* = 18)	Healthy controls (*n* = 18)
Age	30.4 ± 11.2	31.1 ± 10.7
Sex (male/female)	12/6	12/6
Conners’ Adult ADHD Rating Scale—Total score	38.38 ± 7.83*	0.29 ± 0.85*
Conners’ Adult ADHD Rating Scale—Hyperactive/impulsive	20.05 ± 5.81*	0.24 ± 0.66*
Conners’ Adult ADHD Rating Scale—Inattention	18.33 ± 4.74*	0.06 ± 0.24*

An asterisk indicates the statistically significant regions

### DNA methylation of the promoter of SLC6A2

**Fig. 1:**
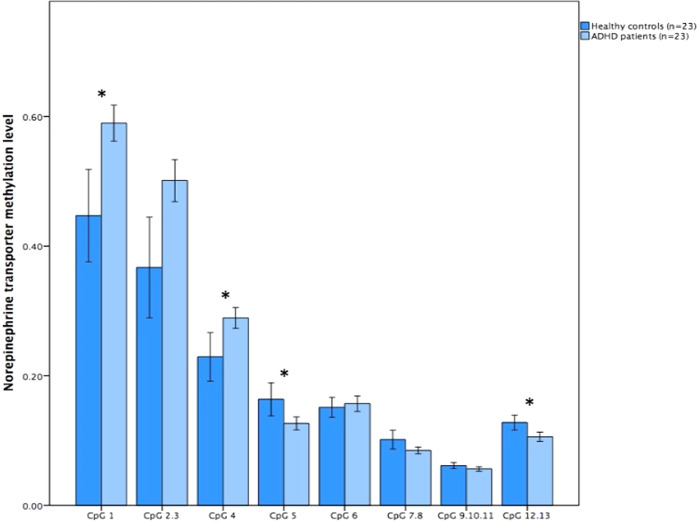
Norepinephrine transporter methylation levels are depicted on the *Y*-axis in different cytosine–phosphate–guanine (CpG) sites across promoter region 1 on the *X*-axis. Light blue represents patients with Attention Deficit Hyperactivity Disorder (ADHD), while darker blue represents healthy controls. An asterisk indicates significant difference between groups after correction for multiple testing (*p* < 0.05)

The rates of methylation levels across regions are comparable to previous studies on SLC6A2 DNA methylation [16, 17]. Toward the 5′ end in promoter region 1, CpG sites were hypermethylated in all subjects, while toward the 3′ end the sites were hypomethylated or marginally methylated. Across CpG sites in promoter region 1 the individual methylation ranged from 9 to 59%. Promoter region 2 is a particularly dense region in terms of CpG sites, several sites were omitted from analysis as they did not fulfil the quality control criteria. In region 2, the individual methylation ranged between 2 and 11% and between 2 and 10% in region 3. Visual inspection of region 1 revealed different levels of methylation, with CpG sites 1, 2.3, and 4 exhibiting relatively high level (methylation > 0.20), whereas CPG sites 5, 6, 7.8, and 12.13 exhibited relatively lower levels (methylation < 0.20), see Fig. 1. Main effects for site (F_45.48_ = 496.24, *p* < 0.001) and group (F_100.41_ = 8.6, *p* = 0.004) were detected for region 1, as well as an interaction between site and group (F_46.39_ = 9.2, *p* < 0.001). For region 1 patients with ADHD had higher methylation levels (0.27) compared with controls (0.22) (Mann–Whitney *U* = 143.5, *p* < 0.007). No difference was found between groups (F_102.62_ = 9.4, *p* = 0.1) in region 2 (Table 3), while in region 3 (F_66.39_ = 2.99, *p* = 0.02) (Table 3), there was a trend for HC to have higher methylation (0.05) than patients (0.04) (Mann–Whitney *U* = 164.5, *p* = 0.03). Examining individual CpG sites for differences between groups the following sites remain statistically different after correction for multiple testing: CpG1, CpG2, CpG4, CpG5, and CpG12.13 across region 1, CpG54.55 in region 2, and CpG1.2 and CpG3.4 in region 3 (Table 3).

**Table 3:** Mean norepinephrine transporter methylation values (mean ± SD) for each site as well as the average for each promoter region.

	ADHD	Controls	*p-*value
Region 1			
CpG 1	0.59 ± 0.06	0.45 ± 0.16	0.001*
CpG 2.3	0.50 ± 0.07	0.37 ± 0.17	0.02
CpG 4	0.29 ± 0.04	0.23 ± 0.09	0.004*
CpG 5	0.13 ± 0.02	0.16 ± 0.05	0.007*
CpG 6	0.16 ± 0.03	0.15 ± 0.04	0.1
CpG 7.8	0.09 ± 0.01	0.10 ± 0.03	0.03
CpG 9.10.11	0.06 ± 0.01	0.06 ± 0.01	0.1
CpG 12.13	0.11 ± 0.02	0.13 ± 0.02	0.002*
Total region 1	0.27 ± 0.03	0.22 ± 0.05	0.007*
Region 2			
CpG 2	0.11 ± 0.03	0.12 ± 0.02	0.1
CpG 3.4	0.07 ± 0.02	0.07 ± 0.01	0.1
CpG 5.6.7	0.06 ± 0.01	0.07 ± 0.03	0.1
CpG 8.9.10	0.05 ± 0.02	0.06 ± 0.03	0.1
CpG 11.12	0.03 ± 0.01	0.03 ± 0.02	0.04
CpG 13	0.03 ± 0.02	0.04 ± 0.04	0.1
CpG 14.15.16	0.10 ± 0.03	0.10 ± 0.01	0.1
CpG 19.20.21	0.03 ± 0.01	0.04 ± 0.01	0.1
CpG 22.23.24	0.06 ± 0.03	0.06 ± 0.02	0.1
CpG 25	0.04 ± 0.03	0.04 ± 0.04	0.1
CpG 26.27	0.07 ± 0.02	0.06 ± 0.02	0.1
CpG 29.30	0.06 ± 0.03	0.06 ± 0.02	0.1
CpG 31.32.33.34	0.05 ± 0.03	0.06 ± 0.03	0.1
CpG 41.42	0.02 ± 0.02	0.02 ± 0.02	0.1
CpG 43.44.45	0.08 ± 0.04	0.08 ± 0.04	0.1
CpG 49.50.51.52	0.05 ± 0.01	0.04 ± 0.01	0.1
CpG 53	0.03 ± 0.01	0.03 ± 0.01	0.1
CpG 54.55	0.02 ± 0.01	0.04 ± 0.05	0.006*
Total region 2	0.05 ± 0.01	0.05 ± 0.02	0.1
Region 3			
CpG 1.2	0.02 ± 0.00	0.04 ± 0.03	0.001*
CpG 3.4	0.02 ± 0.01	0.04 ± 0.03	0.002*
CpG 7	0.03 ± 0.01	0.03 ± 0.01	0.1
CpG 8.9	0.09 ± 0.02	0.11 ± 0.03	0.03
CpG 10	0.02 ± 0.01	0.04 ± 0.03	0.05
CpG 11	0.06 ± 0.02	0.07 ± 0.03	0.1
CpG 12	0.04 ± 0.01	0.05 ± 0.02	0.1
Total region 3	0.04 ± 0.01	0.05 ± 0.02	0.03

An asterisk indicates the statistically significant differences in sites/regions

### Effect of SNPs on methylation

No effects of any of the investigated SNPs were found on either the averaged mean of methylation levels nor on individual CpG site methylation.

### Association of NET methylation with NET BP_ND_

Linear mixed model analysis revealed a main effect of CpG site 4 in region 1 (F_19.99_ = 68.14, *p* < 0.001) as well as an interaction effect of group and CpG site 4 (F_11.30_ = 101.85, *p* < 0.001). Post hoc analysis showed negative correlation between CpG 4 site in patients with ADHD in the following regions: thalamus (*r* = −0.604, *p* = 0.008), locus coeruleus (*r* = −0.510, *p* = 0.03), dorsal raphe nuclei (*r* = −0.614, *p* = 0.007), and medial raphe nuclei (*r* = −0.558, *p* = 0.01) (see Table 4 and Supplemental Figs. 4–7). One potential influential outlier was detected in the locus coeruleus in the patient group. When logtransforming the data, the correlation coefficient increased slightly to *r* = −0.556, *p* = 0.01 (Spearman correlation *r* = −0.581, *p* = 0.01). Removal of outlier resulted in a coefficient of *r* = −0.776, *p* < 0.001. However, no associations between NET methylation and NET BP_ND_ were observed in HC.

**Table 4:** Correlation coefficients (*r*) and *p*-values (*p*) between cytosine–phospate–guanine (CpG) site 4 and the brain regions of interest investigated in patients with Attention Deficit Hyperactivity Disorder (ADHD) and healthy controls.

	ADHD (*n* = 18)	Healthy controls (*n* = 18)
Thalamus	*r* = −0.604	*r* = −0.073
	*p* = 0.008*	*p* = 0.8
Locus coeruleus	*r* = −0.510	*r* = 0.000
	*p* = 0.03	*p* = 1
Dorsal raphe nuclei	*r* = −0.614	*r* = −0.070
	*p* = 0.007*	*p* = 0.8
Medial raphe nuclei	*r* = −0.558	*r* = 0.100
	*p* = 0.01*	*p* = 0.7
Putamen	*r* = 0.175	*r* = −0.096
	*p* = 0.5	*p* = 0.8
Cerebellum	*r* = 0.018	*r* = −0.091
	*p* = 0.9	*p* = 0.7

An asterisk indicates the statistically significant regions

### Association between NET methylation and behavioural scales

**Fig. 2:**
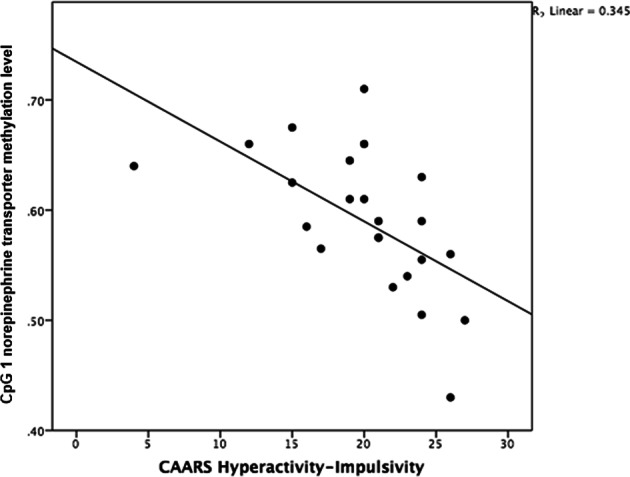
Negative correlation between Conners’ Adult ADHD Rating Scale (CAARS) hyperactivity-impulsivity scale and norepinephrine transporter methylation level in cytosine–phosphate–guanine (CpG) site 1 (*r* = −0.677, *p* = 0.003) in 23 patients with ADHD

**Fig. 3:**
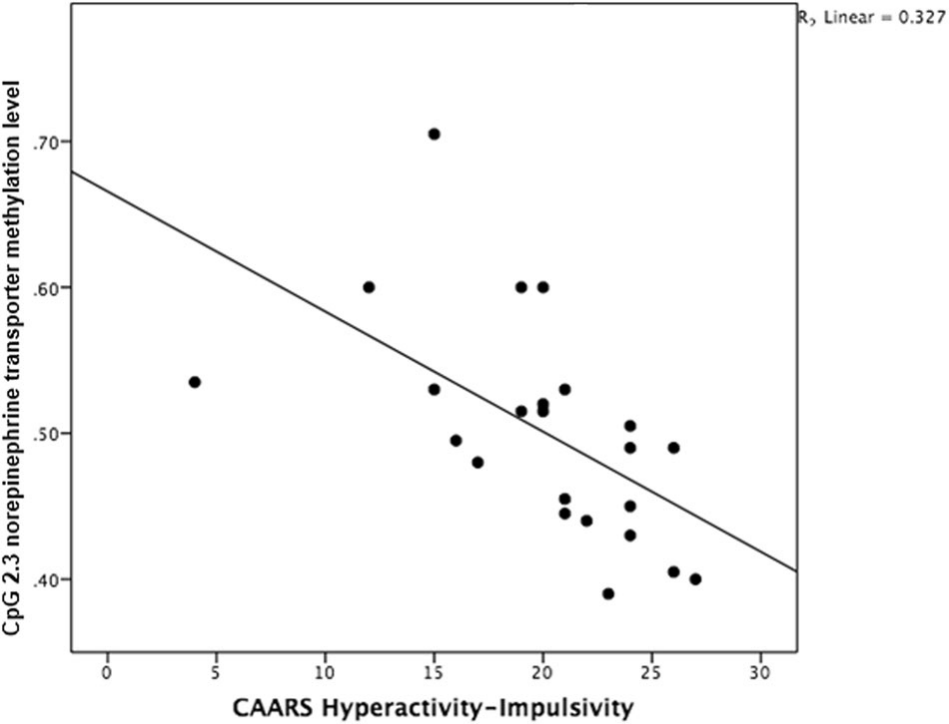
Negative correlation between Conners’ Adult ADHD Rating Scale (CAARS) hyperactivity–impulsivity scale and norepinephrine transporter methylation level in cytosine–phosphate–guanine (CpG) site 2.3 (*r* = −0.609, *p* = 0.006) in 23 patients with ADHD

Possible associations between regions, individual sites, and behavioural symptom scores were explored. Region 1 (*r* = −0.612, *p* = 0.006) and within this region CpG sites 1 (*r* = −0.677, *p* = 0.003) and 2.3 (*r* = −0.609, *p* = 0.006) (see Figs. 2, 3) were negatively associated with hyperactivity–impulsivity scores.

### Combined analysis of SNPs and methylation on NET BP_ND_ and behavior

Lastly, combined effects of genotypes and methylation levels on NET BP_ND_ on one hand and behavioral scales on the other hand were accounted for. No effects withstanding corrections for multiple testing of any of the SNPs investigated in combination with methylation levels on NET BP_ND_ or on behavioral symptoms were found.

## Discussion

In this study, we present the DNA methylation profile of the SLC6A2 gene in patients with ADHD and HC. Our results suggest the differential NET methylation in ADHD to be a promoter region specific. Hypermethylation was detected toward the 5′ end of the promoter in patients with ADHD compared with controls, while this effect reversed toward the 3′ end. Negative association was detected between hyperactivity–impulsivity symptom scores with NET methylation levels for several CpG sites. In a subgroup analysis, we demonstrate for the first time a negative correlation between methylation of a single CpG site with in vivo NET expression in several brain regions in patients only.

In promoter region 1, hypermethylation at the 5′ end was detected in patients in comparison with controls indicating decreased transcriptional activity of the NET. Several potential mechanisms may come at play here. The family of DNA methyltransferases (DNMTs) enzymes are involved in the transfer of methyl groups to DNA. DNMTs may recruit histone deacetylase and histone methylase resulting in transcriptional repression. Secondly, DNA methylation can directly decrease expression by preventing transcriptional factors from binding to the DNA. Thirdly, DNA methylation can repress transcriptional elongation caused by reduced RNA polymerase II occupancy and chromatin accessibility over the gene body. Lastly, methyl-CpG-binding proteins (MBPs) can identify methylated DNA and recruit corepressors in order to silence the transcription and alter the surrounding chromatin [26, 27]. Of the MBPs, the methyl-CpG-binding protein 2 (MeCP2) is perhaps the most studied and a key transcriptional regulator associated with transcriptional repression. It binds to methylated DNA and recruits other factors that alter the chromatin structure [28]. NET is hypothesized to be repressed in human disorders where DNA hypermethylation has been demonstrated using peripheral whole blood such as in panic disorder and cardiovascular disease [29, 30]. In the study of Esler et al., it was evident that MeCP2 binds to the methylated promoter region of the NET in panic disorder patients [30]. Another investigation found MeCP2 expression levels to be significantly decreased in boys with ADHD [31]. It is therefore likely that the MeCP2 binds distinctively to methylated regions of the NET in ADHD patients. Furthermore, it can be assumed that MeCP2 binds distinctively to hypermethylated regions of the NET promoter resulting in extended repression of NET expression in ADHD patients. The MeCP2 is though not exclusively bound to methylated DNA. It has previously been determined that it also binds to hypomethylated sites in the promoter region of the NET [15, 16]. For certain sites in promoter region 1 and 3 we found the effect to be reversed for patients, they had lower methylation levels in comparison with controls, which potentially could be explained by the multifunctional role of the MeCP2 of it being able to bind to hypomethylated regions. This is however up to speculation and requires further research in order to unravel the underlying mechanism.

Interestingly, we detected negative association between methylation of a single CpG and NET expression in several brain regions of interest. Higher methylation levels were associated with lower in vivo expression of the NET in the thalamus, locus coeruleus, and the raphe nuclei. This finding supports previous evidence of the molecular effect of DNA methylation on expression [27, 32]. Strikingly, this was only observed in patients and not in HC. In addition to the potential effects of the before mentioned epigenetic factors, other factors may come into play here. As the SLC6A2 has many *cis*-regulatory elements in its promoter region it is possible that they behave distinctively in ADHD. The *cis*-regulatory transcription factor nuclear factor kappa B (NF-кB) is a part of that particular CpG site, possibly affecting the transcription in patients [33]. The exact mechanism of action is up to speculation, however a previous study has shown that inhibition of NF-кB significantly upregulates the NET [34]. Although NF-кB is a key regulator in inflammatory response it has also been shown to be involved in synaptic plasticity, memory, stress, addiction, and locomotor activity [35]. The NET is well established for its role in memory and stress [3] and is also implicated to modulate synaptic plasticity [36]. It is plausible that transcription at this particular site differentially affects patients with ADHD depending on the amount of exposure to environmental factors. Studies have implicated the role of oxidative stress [37, 38], stress, smoking, alcohol, and other pre- and perinatal risk factors for ADHD [39, 40]. Future studies should therefore consider other various risk factors in order to get a clearer picture of the underlying pathophysiology.

We found several sites within promoter region 1 to be negatively associated with symptoms of hyperactivity and impulsivity. More precisely, lower methylation levels were associated with increased symptom severity. Decreased methylation levels may represent higher transporter expression resulting in increased uptake of extracellular norepinephrine. This is of particular importance as norepinephrine modulates multiple cognitive processes, including inhibitory control, that are often impaired in ADHD. Furthermore, common medication for ADHD such as MPH and atomoxetine significantly improve clinical symptoms such as hyperactivity by blocking uptake of the NET and increasing NE levels [41–43]. Our results are in line with our previous study where we found hyperactivity–impulsivity scores to be genotype dependent. We found patients carrying the major allele of rs40615 and rs15534 to have higher scores and higher NET availability [9]. Our results are also transferable to studies on the dopamine transporter (DAT1) as medications for ADHD target these two systems by increasing levels of dopamine and norepinephrine. One study found a correlation between the DAT1 gene and symptom responses of hyperactivity–impulsivity following MPH treatment. They found that less methylation was associated with greater MPH response [12]. Another study detected negative association between DAT1 methylation and scores of hyperactivity [44].

We can only speculate about the differential association found between certain CpG sites with either symptomology or in vivo expression. Firstly, as the NET has several regulatory elements and transcription factor binding sites within the gene it is possible they behave in a distinct way having different consequences on behavior or brain function. Secondly, the effects may be too small to detect due to the sample size. Third option is the possible influence of polymorphisms located on the gene possibly affecting or interacting with epigenetic mechanisms resulting in a certain phenotype.

We failed to demonstrate any effect of SNPs on methylation levels or any associations between methylation levels and NET binding to be genotype dependent, suggesting that the epigenetic effect is stronger and independent of genotypic variation. We can however not rule out any potential effects of genetic variation on the methylation levels as the sample size in the subgroup analysis is quite small. Moreover, no combined effects of methylation levels and genotypes on brain binding potentials or on behavioral scales was found, further emphasizing the need for future testing using larger sample sizes. Furthermore we only investigated a handful of SNPs that we had previously shown to have a genotype dependent effect on the NET binding [9].

We must acknowledge several limitations to our study. Firstly, as with many neuroimaging studies, the sample size is considered quite small, thus replications in larger samples are warranted. Secondly, we can only estimate DNA methylation of the NET from whole blood as a proxy for the brain, but DNA methylation tends to be tissue specific [45]. We cannot draw definite conclusions about the methylation patterns in the brain although using peripheral blood is considered to be feasible as several studies have shown correlations between peripheral markers and the brain [46]. Last, although we did a new analysis and were able to confirm our previous results and successfully increase our call rates, we were unable to do the analysis using a different method. Further studies using different methods such as pyrosequencing are necessary in order to validate our results. On the other hand, the pattern of methylation observed within regions is in line with the study by Bayles et al. [17].

Regardless of our limitations, we give rise to new insights of the role of epigenetic mechanisms underlying NET imbalance in ADHD. We demonstrate for the first time differential DNA methylation levels in the SLC6A2 between patients with ADHD and HC. Differential methylation in patients may possibly be due to transcription factors behaving in a distinct manner in ADHD. Higher site-specific methylation at CpG 4 seems to predict in vivo availability in a region specific manner and lends support to altered transcriptional control in ADHD. We show an epigenetic effect of DNA methylation on behavioural control for several sites, namely hyperactivity–impulsivity symptoms. While these results look promising, future studies are required, including larger sample sizes and genetic variants covering the whole region of the NET gene. Furthermore, although not detected in this study, future research should also include patients currently undergoing pharmacotherapy as it may affect the DNA methylation [47, 48].

## Supplementary information

Supplemental material
