# Supplementary material for: Association of norepinephrine transporter methylation with in vivo NET expression and hyperactivity–impulsivity symptoms in ADHD measured with PET
Source: Mol Psychiatry. 2019 Aug 5;26(3):1009–18. doi: 10.1038/s41380-019-0461-x (PMC7910214; doi:10.1038/s41380-019-0461-x)
Supplement: Supplementary file 1 — Supplemental material [file 41380_2019_461_MOESM1_ESM.docx]

| Supplemental table I. Primer sequences | | | | |
| --- | --- | --- | --- | --- |
| Assay | Start^1^ | Size^2^ | Left Primer | Right Primer |
| NET_01 | 55655473 | 290 | TGTTGGGTTGTGATTTGTTAATATG | CCAAAAACACCAAACCTCTAATCT |
| NET_07 | 55655750 | 520 | AGATTTTAGTTTTGGAGAGTTTGTTA | CTCCCAAACAAACCTAACCCTAT |
| NET_08 | 55656191 | 109 | GATAGGGTTAGGTTTGTTTGGGA | CAACAACCACAAAAACCTTCT |
| NET_22 | 55656191 | 189 | GATAGGGTTAGGTTTGTTTGGGAG | CCCCAAATAACTAAAATTAAACTCACC |
| ^1^Start of target sequence, position according to GRCh38.p7; ^2^Size of PCR fragments [bp] | | | | |

*Additional analysis - Effect of SNPs on NET BP_ND_ and behavior*

Analysis revealed an interaction between group and the SNP rs28386840 in the thalamus (F_9.06_=32.00, p=0.005) as well as for the SNP rs2242446 in the thalamus (F_8.68_=28.55, p=0.006). Post hoc analysis showed that major allele carriers had lower binding in comparison to minor allele carriers in healthy controls only (*t=* -3.25, p=0.005, rs28386840) as well as (*t=* -3.25, p=0.005, rs2242446). Furthermore, healthy controls carrying the major allele had lower binding than patients carrying the major allele (*t=* -2.80, p=0.012, rs28386840) and (*t=* -2.78, p=0.013, rs2242446).

An interaction between the SNP rs15534 and group was detected in the cerebellum (F_6.86_=30.86, p=0.014) as well as for the SNP rs40615 (F_5.90.68_=29.18, p=0.021). Post hoc t tests revealed higher binding in major allele carriers in healthy controls compared to patients for the SNPs rs15534 (*t=* 3.01, p=0.007) and for the SNP rs40615 (*t=* 3.06, p=0.007).

Potential effects on behavioral scales were tested for between genotype groups in patients only. Before applying correction for multiple comparisons, genotype dependent correlation was found for the SNP rs15534 between cerebellar norepinephrine transporter (NET) binding potential (BP_ND_) and CAARS hyperactivity/impulsivity scores. Major allele (C) carriers (n=10) showed a positive correlation with behavioral symptoms (r=0.698, p=0.025) and showed a negative correlation for the minor allele (CT+T) carriers (n=8) (r=-0.667, p=0.05) (supplemental figure 2). Additionally, similar effect was found for the SNP rs40615. Positive correlation for major allele (C) carriers (n=9) with behavioral symptoms (r=0.743, p=0.02) was detected while the minor allele (CT+T) carriers (n=9) showed a negative association (r=-0.658, p=0.05) (supplemental figure 3).

Further analysis failed to detect any main effects or interactions between the other brain regions of interest, group and SNPs on binding potentials.

**Supplemental table II.** Mean and standard deviations depending on genotype is given for NET BP_ND_ values in the investigated brain

regions of interest in patients with ADHD and controls

|  | rs28386840  (A/T) | rs2242446  (T/C) | rs15534  (C/T) | rs40615  (T/A) |
| --- | --- | --- | --- | --- |
| Thalamus (ADHD)  Thalamus (HC) | 0.50±0.10/0.48±0.03  0.39±0.06/0.52±0.14 | 0.51±0.11/0.47±0.04  0.39±0.07/0.52±0.14 | 0.51±0.08/0.47±0.09  0.47±0.14/0.47±0.11 | 0.52±0.09/0.47±0.08  0.46±0.15/0.47±0.08 |
| Locus coeruleus (ADHD)  Locus coeruleus (HC) | 0.41±0.13/0.42±0.11  0.32±0.11/0.43±0.15 | 0.41±0.14/0.41±0.10  0.32±0.11/0.42±0.15 | 0.41±0.10/0.41±0.14  0.37±0.17/0.40±0.07 | 0.41±0.11/0.41±0.13  0.37±0.17/0.40±0.07 |
| Dorsal raphe nuclei (ADHD)  Dorsal raphe nuclei (HC) | 0.46±0.14/0.45±0.13  0.31±0.18/0.48±0.14 | 0.46±0.15/0.45±0.11  0.31±0.18/0.48±0.14 | 0.47±0.14/0.44±0.13  0.37±0.19/0.47±0.15 | 0.47±0.14/0.44±0.12  0.36±0.20/0.46±0.14 |
| Medial raphe nuclei (ADHD)  Medial raphe nuclei (HC) | 0.51±0.15/0.53±0.17  0.41±0.14/0.54±0.19 | 0.52±0.17/0.52±0.15  0.41±0.14/0.54±0.19 | 0.52±0.14/0.52±0.18  0.47±0.21/0.51±0.09 | 0.52±0.15/0.52±0.17  0.47±0.22/0.51±0.08 |
| Putamen (ADHD)  Putamen (HC) | 0.19±0.05/0.16±0.04  0.17±0.04/0.18±0.04 | 0.19±0.05/0.16±0.04  0.17±0.04/0.18±0.04 | 0.18±0.04/0.18±0.06  0.18±0.04/0.17±0.04 | 0.18±0.04/0.18±0.06  0.17±0.05/0.17±0.03 |
| Cerebellum (ADHD)  Cerebellum (HC) | 0.19±0.10/0.21±0.09  0.22±0.06/0.26±0.07 | 0.19±0.10/0.21±0.09  0.22±0.06/0.26±0.06 | 0.16±0.06/0.24±0.11  0.25±0.06/0.22±0.06 | 0.16±0.06/0.23±0.11  0.26±0.05/0.20±0.07 |


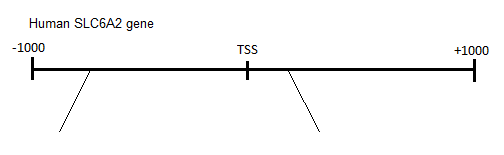


GCTAAGCC^1^GCTCAAACAAAACTGCCCAACAGCCC^2^GCTGGCC^3^GCCTATTTGCAGCACTGGGCCCTGAGCC^4^GCACATTCCCATTTC^5^GTTGATAAAGAAACTGACCAGATAGTTTAAGTGGCCTGCTGC^6^GGAAGACAGAGCTGGTGCTGCACC^7^GGTC^8^GCTGCTTCCCCAGTCCTTTTTTGGCCTCCTTTCTGAC^9^GC^10^GAC^11^GCAGACCCCAGTTCTGGAGAGTCTGTCACTC^12^GCTCCCC^13^GTGGTGGGAGATCAGAGGCCTGGTGTCCTTGGGAGCGGCGAGC^5^GGTGCTC^6^GGC^7^GCAGGATAGAAAGGGAGTGC^8^GC^9^GCCC^10^GAGTCCCCCAGATCCCTGGGAACCC^11^GC^12^GCCACCCTCCC^13^GCCCCTGCCCATCCCC^14^GGCC^15^GC^16^GCTGTCAGTCTCCATTAGCGCTAACAGGCTCCAGACGGAGC^19^GGGCC^20^GGGC^21^GCTGGGTTAATGCAATC^22^GGC^23^GC^24^ GTTACCTGGGGC^25^GCAGGCTACATTACCAGCCC^26^GGCCCCC^27^GCCAGGCACGGCCAGAACCAGTCAGCCC^29^GC^30^GCCCTGCC^31^GGCC^32^GCCCC^33^GC^34^GCCTCCAGCTCTTCCCCGGCCCCGCCCGAACGCCACACGGCGGAGCCCAGCCCCAGCCC^41^GC^42^GCCCTAGAGCCTGCCAAGGC^43^GCC^44^GCC^45^GGTCGGGGGCCGGCAGGGCGCAAGGCACCAGGGATCCCCTC^49^GCC^50^GCC^51^GGACAC^52^GTGAGTGC^53^GCCCTGAGC^54^GC^55^GGGACAGGGCTAGGTCTGCCTGGGAGGCCC^1^GGGCC^2^GAGAC^3^GC^4^GCCAGCAGAGGGCTAGCGAGTTTGTAGTGCAGTGACGTTAAGTGTCC^7^GAGAAGGCTCCTGTGGCTGTTGAAGTGTC^8^GC^9^GGACCTGAGCTTGGGGAGGGGGTC^10^GGCAC^11^GCTGCCCTCAGCCTC^12^GGTGAGT

**Supplemental figure 1.** Genomic sequence of the promoter region of the SLC6A2 gene extending into exon 1 for the three regions analysed. Location of the sequence is chr16:55655473-55656461. Highlighted sequences represent regions 1-3. Number of each site corresponds to sites reported in the manuscript. TSS stands for transcription start site.


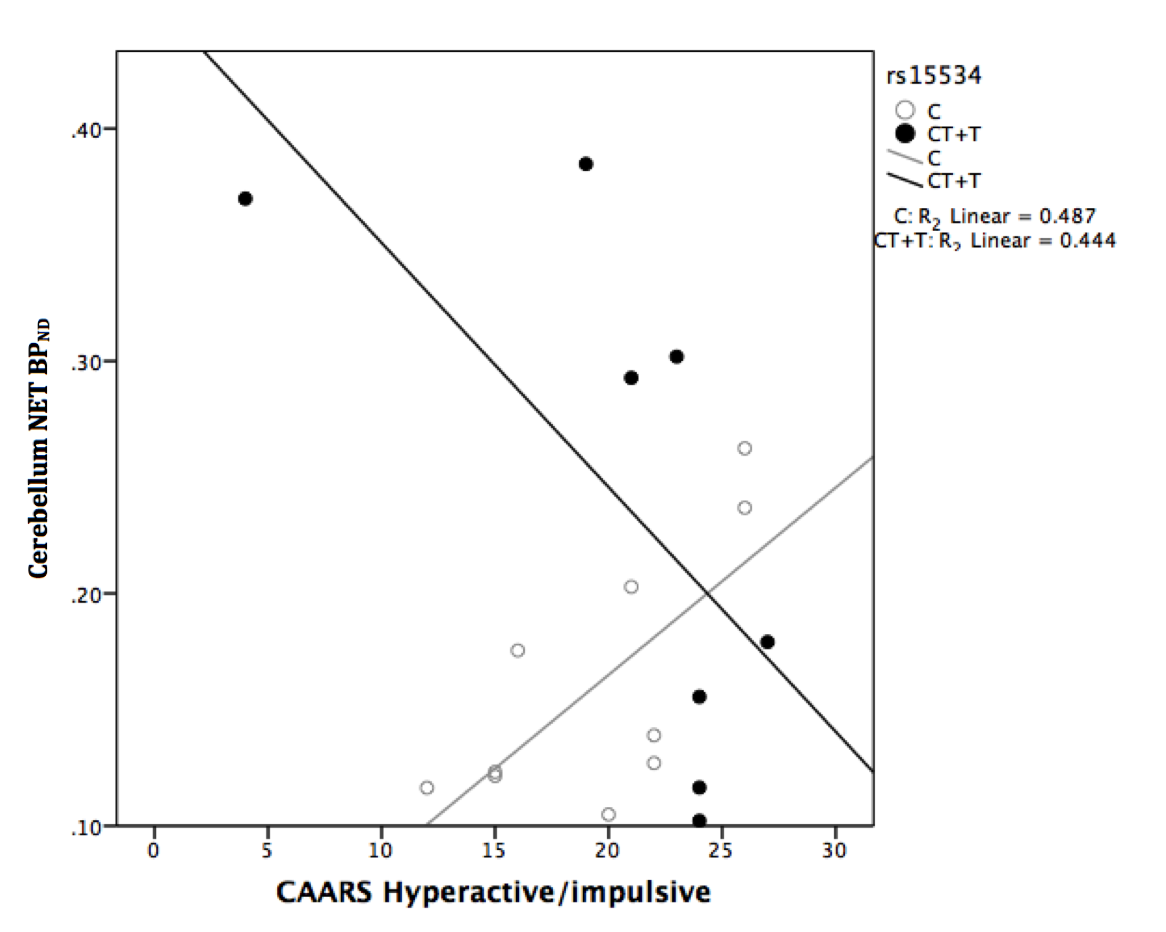


**Supplemental figure 2.** Genotype dependent correlation between cerebellar norepinephrine transporter (NET) binding potential (BP_ND_) and CAARS hyperactivity/impulsivity scores for the SNP rs15534. Major allele (C) carriers (n=10) shows a positive correlation with behavioral symptoms (r=0.698, p=0.025) while the minor allele (CT+T) carriers (n=8) showed a negative association (r=-0.667, p=0.05).


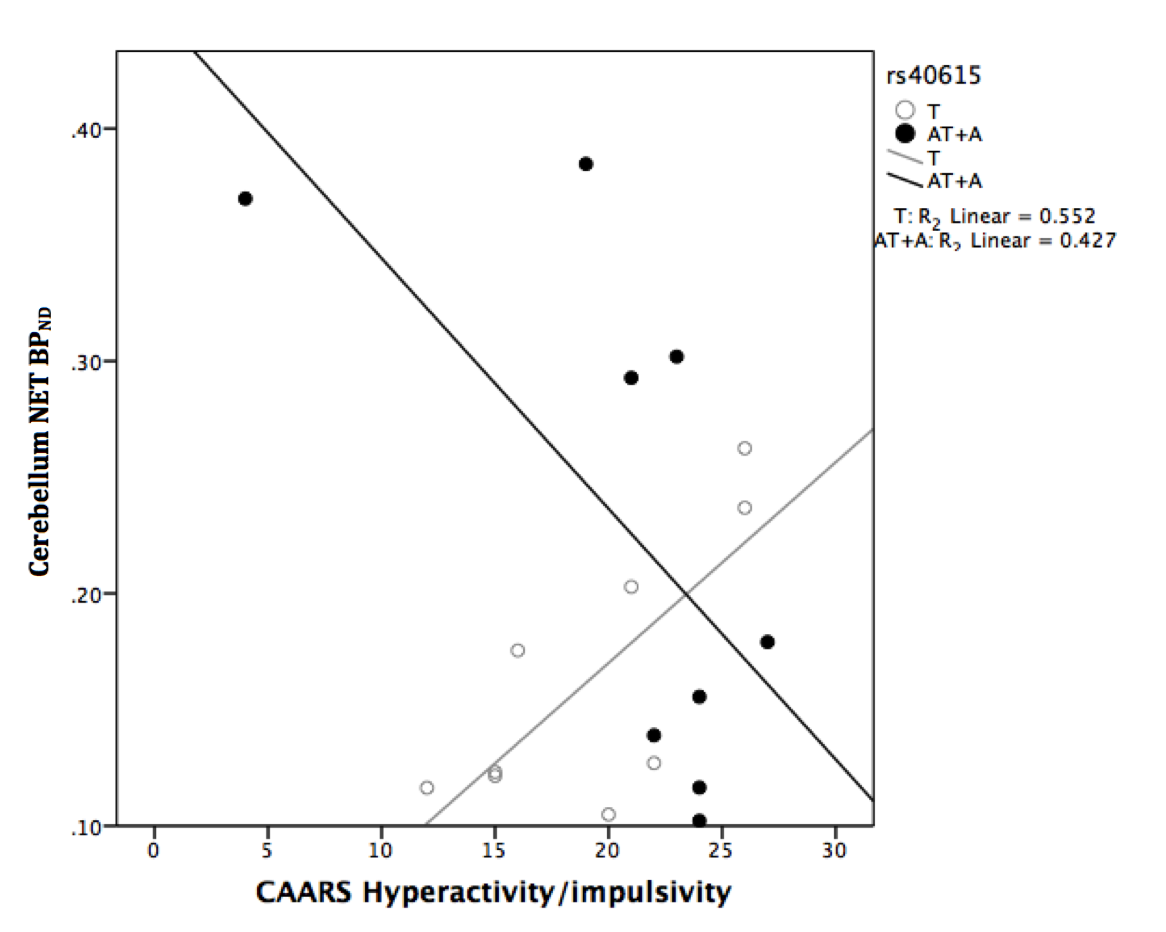


**Supplemental figure 3.** Genotype dependent correlation between cerebellar norepinephrine transporter (NET) binding potential (BP_ND_) and CAARS hyperactivity/impulsivity scores for the SNP rs40615. Major allele (C) carriers (n=9) shows a positive correlation with behavioral symptoms (r=0.743, p=0.02) while the minor allele (CT+T) carriers (n=9) showed a negative association (r=-0.658, p=0.05).


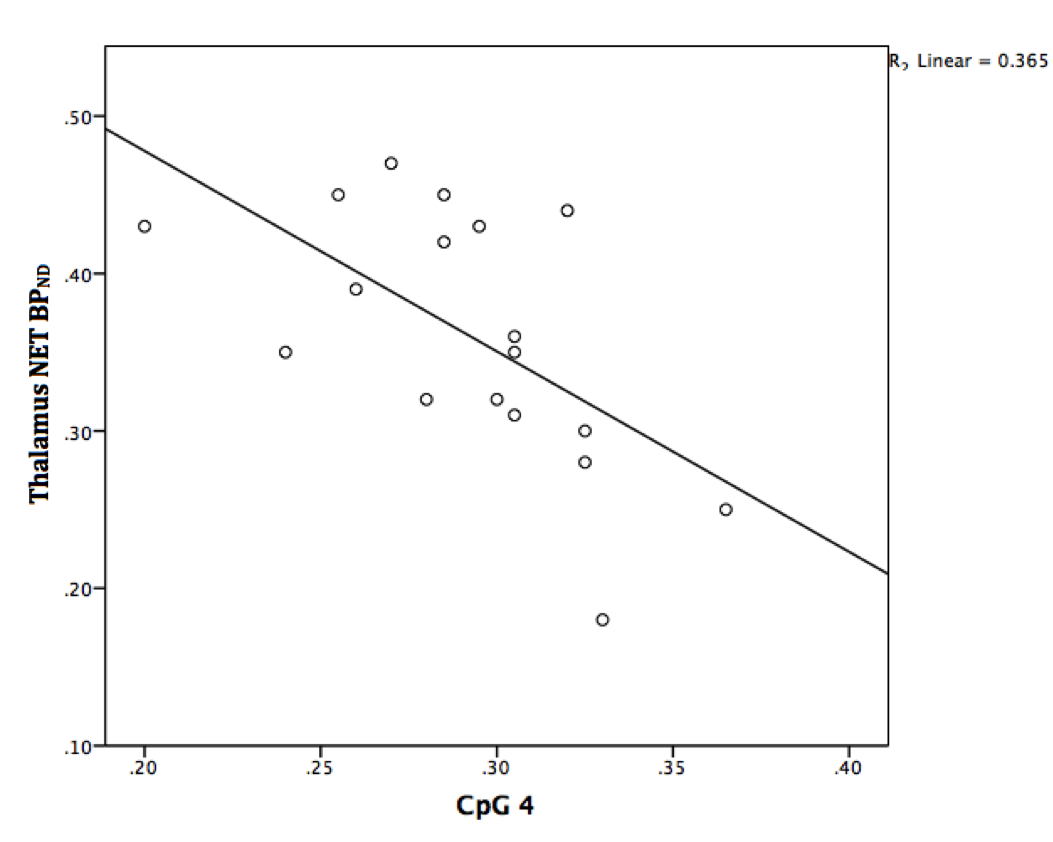


**Supplemental figure 4.** Negative correlation between DNA methylation level at cytosine-phosphate-guanine (CpG) site 4 and thalamus norepinephrine transporter (NET) binding potential (BP_ND_) in patients with Attention Deficit Hyperactivity Disorder (ADHD) (r=-0.604, p=0.008).


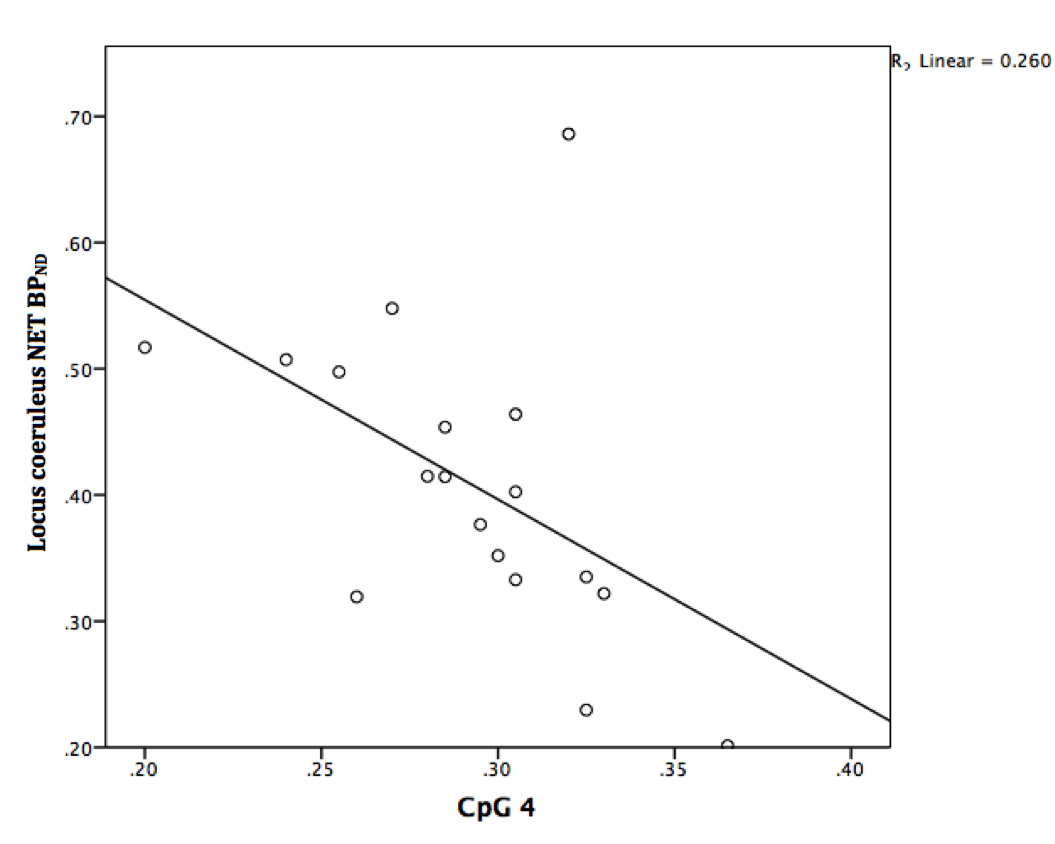


**Supplemental figure 5.** Negative correlation between DNA methylation level at cytosine-phosphate-guanine (CpG) site 4 and locus coeruleus norepinephrine transporter (NET) binding potential (BP_ND_) in patients with Attention Deficit Hyperactivity Disorder (ADHD) (r=-0.510, p=0.03).


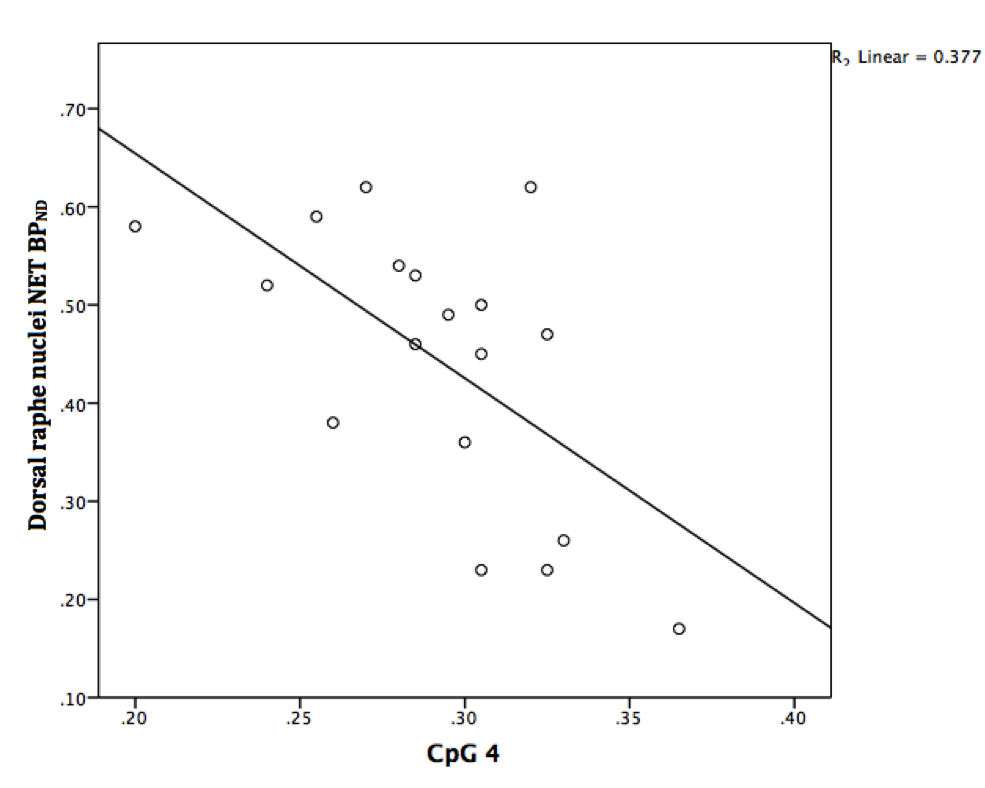


**Supplemental figure 6.** Negative correlation between DNA methylation level at cytosine-phosphate-guanine (CpG) site 4 and dorsal raphe nuclei norepinephrine transporter (NET) binding potential (BP_ND_) in patients with Attention Deficit Hyperactivity Disorder (ADHD) (r=-0.614, p=0.007).


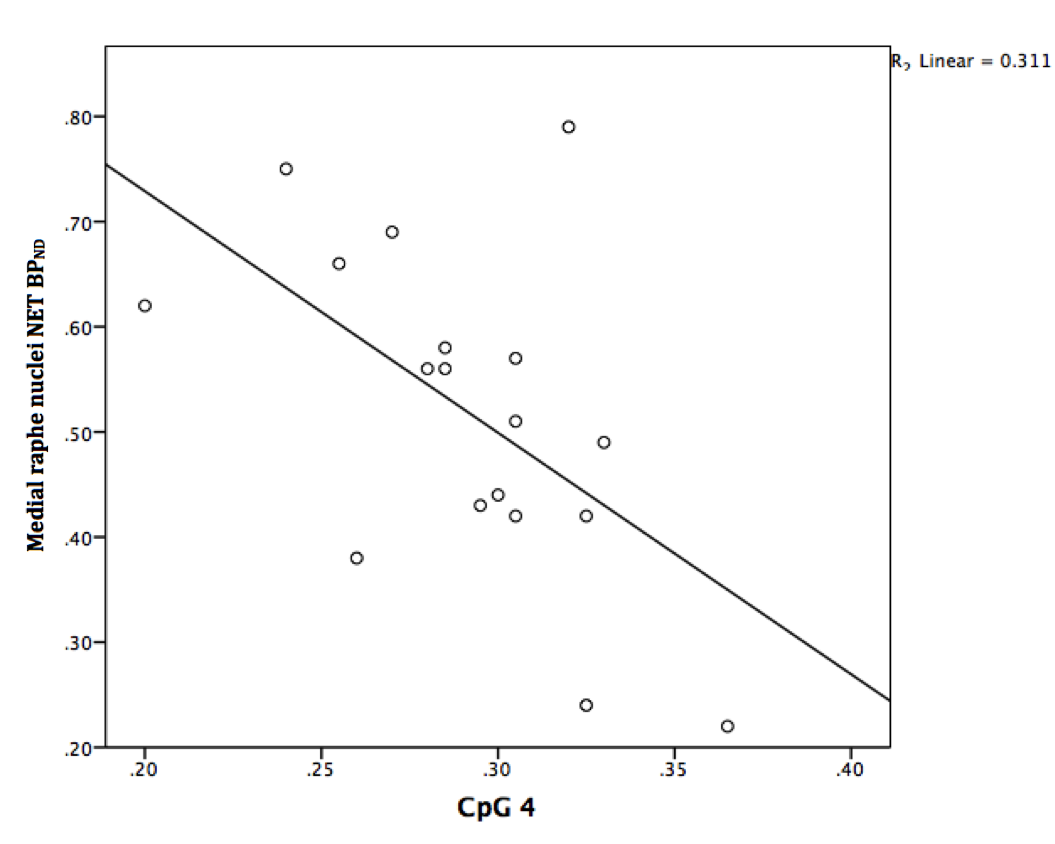


**Supplemental figure 7.** Negative correlation between DNA methylation level at cytosine-phosphate-guanine (CpG) site 4 and medial raphe nuclei norepinephrine transporter (NET) binding potential (BP_ND_) in patients with Attention Deficit Hyperactivity Disorder (ADHD) (r=-0.558, p=0.01).
